# Inter-Tissue Gene Co-Expression Networks between Metabolically Healthy and Unhealthy Obese Individuals

**DOI:** 10.1371/journal.pone.0167519

**Published:** 2016-12-01

**Authors:** Lisette J. A. Kogelman, Jingyuan Fu, Lude Franke, Jan Willem Greve, Marten Hofker, Sander S. Rensen, Haja N. Kadarmideen

**Affiliations:** 1 Department of Large Animal Sciences, Faculty of Health and Medical Sciences, University of Copenhagen, Frederiksberg, Denmark; 2 University of Groningen, University Medical Center Groningen, Department of Genetics, Groningen, The Netherlands; 3 University of Groningen, University Medical Center Groningen, Department of Paediatrics, Groningen, The Netherlands; 4 Department of General Surgery, Atrium Medical Center Parkstad, Heerlen, the Netherlands; 5 Pediatrics Molecular Genetics, University of Groningen, University Medical Center Groningen, Department of Genetics, Groningen, The Netherlands; 6 Department of Surgery, NUTRIM School of Nutrition and Translational Research in Metabolism, Maastricht University, Maastricht, The Netherlands; Universidad Pablo de Olavide, SPAIN

## Abstract

**Background:**

Obesity is associated with severe co-morbidities such as type 2 diabetes and nonalcoholic steatohepatitis. However, studies have shown that 10–25 percent of the severely obese individuals are metabolically healthy. To date, the identification of genetic factors underlying the metabolically healthy obese (MHO) state is limited. Systems genetics approaches have led to the identification of genes and pathways in complex diseases. Here, we have used such approaches across tissues to detect genes and pathways involved in obesity-induced disease development.

**Methods:**

Expression data of 60 severely obese individuals was accessible, of which 28 individuals were MHO and 32 were metabolically unhealthy obese (MUO). A whole genome expression profile of four tissues was available: liver, muscle, subcutaneous adipose tissue and visceral adipose tissue. Using insulin-related genes, we used the weighted gene co-expression network analysis (WGCNA) method to build within- and inter-tissue gene networks. We identified genes that were differentially connected between MHO and MUO individuals, which were further investigated by homing in on the modules they were active in. To identify potentially causal genes, we integrated genomic and transcriptomic data using an eQTL mapping approach.

**Results:**

Both *IL-6* and *IL1B* were identified as highly differentially co-expressed genes across tissues between MHO and MUO individuals, showing their potential role in obesity-induced disease development. WGCNA showed that those genes were clustering together within tissues, and further analysis showed different co-expression patterns between MHO and MUO subnetworks. A potential causal role for metabolic differences under similar obesity state was detected for *PTPRE*, *IL-6R* and *SLC6A5*.

**Conclusions:**

We used a novel integrative approach by integration of co-expression networks across tissues to elucidate genetic factors related to obesity-induced metabolic disease development. The identified genes and their interactions give more insight into the genetic architecture of obesity and the association with co-morbidities.

## Introduction

Obesity, characterized by an excessive accumulation of adipose tissue in the body, has major consequences for human health, like type 2 diabetes (T2D) and nonalcoholic steatohepatitis (NASH). However, it has now been acknowledged that (extremely) obese individuals may also be metabolically and cardiorespiratory fit, so called metabolic healthy obese (MHO) [1, 2]. It is estimated that 10 − 25 percent of the obese individuals are MHO [1]. The excessive accumulation of adipose tissue in case of obesity disturbs the endocrine balance. Already in 1968, it was indicated that the functioning of insulin metabolism is dependent upon adipose cell size and that adaptive functioning of adipose cells is linked to the metabolic condition of individuals [3]. The expandability of the adipose tissue to be able to store large amounts of fat may be an important factor determining obesity-induced metabolic disturbances [4]. However, expandability is not an unlimited process; in fact, adipose tissue storage capacity may become saturated, resulting in excess of fat “overspilled” to non-adipose tissues and subsequent lipotoxicity which can lead to metabolic syndrome [5]. In such cases, obesity results in elevated levels of free fatty acids (FFA) affecting the pancreatic beta cells, and in the secretion of a group of adipose tissue derived cytokines, the adipokines. The direct effect of FFA is thought to be the result of activation of multiple intracellular signals in the beta cell, eventually leading to apoptosis and reduced insulin secretion [6]. To date, involved genetic factors and pathways are largely unknown.

Here, we aim to find genetic and molecular mechanisms important for human obesity-induced disease development by comparing inter-tissue gene co-expression of MHO and metabolically unhealthy obese (MUO) individuals. A study in mice showed important dynamic inter-tissue crosstalk in obesity development, with a key role for inflammatory pathways [7]. Also, the inter-tissue crosstalk of adipose tissue and skeletal tissue has shown its importance in obesity [8] and insulin resistance [9]. Weighted Gene Co-expression Network Analysis (WGCNA) [10] clusters genes based on gene-gene interactions and is used to unravel genetic mechanisms of complex diseases, including obesity [11–14]. We here use this approach to create an inter-tissue network, giving the potential of studying the inter-tissue gene co-expression and hereby gaining insight into the genetic architecture across tissues and pathogenesis of obesity and comorbidities. This approach has previously led to the successful identification of key drivers of coronary artery disease [15]. In this study, we investigate the co-expression patterns of insulin-related genes in severely obese individuals, some of whom are MHO. The expression profiles of four tissues (liver, muscle, subcutaneous adipose tissue, and visceral adipose tissue) are investigated using a novel integrative inter-tissue approach. We furthermore look for potential causal genetic variants for metabolic differences under similar obesity state, by integrating, modeling, and analyzing genomic and transcriptomic data by an eQTL approach.

## Materials and Methods

### Genomic and transcriptomic data from the study population

#### Subjects

In this study, 60 severely obese individuals (42 males, 18 females) who underwent elective bariatric surgery were included. All individuals were free from acute or chronic inflammatory or degenerative diseases, and did not consume a high amount of alcohol (>10 g/day) or anti-inflammatory drugs. This study was approved by the Medical Ethical Board of Maastricht University Medical Centre, in line with the guidelines of the 1975 Declaration of Helsinki. Informed consent in writing was obtained from each subject personally. All subjects were deeply phenotyped for a number of metabolic measures, e.g. systemic glucose, cholesterol, and C-reactive protein concentrations.

#### Expression profiling

During elective bariatric surgery samples were taken from the liver, muscle (*musculus rectus abdominis*), subcutaneous adipose tissue (SAT; abdominal), and visceral adipose tissue (VAT; *omentum majus*). RNA was hybridized to Illumina HumanHT12 BeadChips and scanned on the Illumina BeadArray Reader. Raw intensity data were extracted using Illumina’s BeadStudio Gene expression module v3.2. The samples were not pooled. All raw data are publicly available at Gene Expression Omnibus (GSE22070). Raw expression data were normalized by performing a quantile normalization and log2 transformation.

#### Genotyping

After eight hours of fasting on the morning of elective bariatric surgery, venous blood samples were obtained. DNA was extracted and genotyped using HumanOmniBeadChips (Illumina) and imputed using the GIANT release from the 1000 Genomes project (5,763,069 unique SNPs).

More information about the study population, expression profiling, and genotyping can be found in previous publications [16–19].

### Filtering of insulin-related genes for inter-tissue network construction

Insulin-related genes were filtered from the expression dataset. First, GO-terms related to insulin were found using AmiGO 2 (http://geneontology.org), by using the search term “insulin”, resulting in the detection of 92 GO-terms. We then used Biomart [20] to detect genes that have been associated with those GO-terms, resulting in 648 unique genes corresponding to 885 transcripts.

### Inter-tissue behavior of individual genes

Potential biologically important inter-tissue genes are expected to show altered inter-tissue co-expressions, and were therefore identified by the difference of the inter-tissue co-expression between the MHO and MUO subnetworks. To identify those important inter-tissue genes, we calculated the correlation between tissues for each insulin-related gene in two subnetworks (MHO and MUO). This resulted in a correlation between each tissue-pair (liver-muscle, liver-SAT, liver-VAT, muscle-SAT, muscle-VAT, SAT-VAT) for each insulin-related gene in both the MHO and MUO subnetwork. Following this, between each tissue-pair the absolute difference between MHO and MUO subnetworks was calculated for each gene. Then, the sum of tissue-pair differences (inter-tissue co-expression) in both networks was calculated.

### Inter-tissue co-expression network analysis

**Fig 1:**
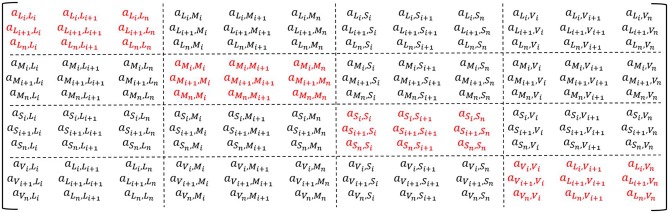
The adjacency matrix to construct the within- and inter-tissue gene network. The matrix presents the adjacency for L = Liver, M = Muscle, S = Subcutaneous Adipose Tissue, V = Vat, for *i* till *n* genes. In red the within-tissue blocks, in black the inter-tissue blocks.

To gain insight in gene-gene interactions we constructed weighted gene co-expression networks, using the Weighted Gene Co-expression Network Analysis (WGCNA) R-package [10]. The networks were constructed across tissues for both MHO and MUO individuals by calculating Pearson’s correlation coefficients between each gene-pair across all tissues. We created the adjacency matrix with diagonal blocks representing within-tissue correlations (tissue-specific) and the off-diagonal blocks representing inter-tissue co-expression (Fig 1). The inter-tissue co-expression was computed by taking the i^th^ gene in the t^th^ tissue and correlated with all other G_i_ genes in a T_t_ different tissue and repeated for all genes in G and all tissues in T. Here, T is a vector with the number of tissues and G a vector with the number of genes in a given tissue. The mean and standard deviation of each block showed no significant difference between the within-tissue and inter-tissue blocks, showing no biasing difference in connection strength between within-tissue blocks and inter-tissue blocks. Modules were detected by first calculating the dissimilarity Topological Overlap Measure (TOM), which was used for clustering. Modules were defined as branches of the cluster dendrogram, which were detected using the Dynamic Tree Cut library for R [21], with a minimum module size of 50 genes.

### eQTL mapping

The eQTL mapping was performed using the eQTL-mapping-pipeline (v.1.2.4) developed by the Department of Genetics, University Medical Centre Groningen, The Netherlands, which can be found at http://github.com/molgenis/systemsgenetics/tree/master/eqtl-mapping-pipeline [22]. We used previously normalized expression data of the selected insulin-genes, as described above. The SNPs were filtered based on call rate (> 0.95), Hardy-Weinberg equilibrium (P > 1E^-4^), and minor allele frequency (MAF > 0.05). We performed the cis-eQTL analysis, whereby the distance between SNP and probe was set on 1 Mb on either side of the probe. Detected p-values were corrected for multiple-testing by permutation testing (n = 10), and eQTLs were considered to be significant with a FDR < 0.05. The eQTL mapping was performed with the expression data of each tissue.

### Functional annotation and visualization

Genes in detected modules were retained for gene set enrichment analysis (GSEA) based on their Module Membership (MM; correlation of gene with module eigengene). GSEA was performed using HumanMine (http://humanmine.org), detecting overrepresented GO-terms and KEGG pathways. Visualization of modules was performed in Cytoscape [23].

## Results and Discussion

All individuals in this study were severely obese (BMI > 35) and underwent bariatric surgery. Deep metabolic phenotyping resulted in an overview of the metabolic state of the individuals, showing that nearly half were MHO (defined as having neither T2D nor NASH). Among the MUO individuals, 18 individuals suffered from T2D (7 males, 11 females), 27 suffered from NASH (8 males, 19 females) and of them, 13 individuals had both T2D and NASH (4 males, 9 females). Descriptive statistics of the metabolic phenotypes showed a significant difference (P < 0.05) between MHO and MUO individuals for glucose, glycated hemoglobin (HbA1c), FFA, and aspartate transaminase (ASAT) (Table 1). Those metabolic phenotypes did not show a significant difference between males and females (P>0.05), though we did find a significant difference for BMI, waist-hip ratio (WH-ratio), insulin, total cholesterol and low-density lipoprotein (LDL) levels (P<0.05). A significant difference in age was found: the MHO individuals were younger than the MUO individuals, which is in agreement with a study that found a decreasing prevalence of metabolic healthy obesity with age [24].

**Table 1:** Descriptive statistics with mean and standard deviation of the individuals.

	All	MHO	MUO	P-value
**Number**	60	28	32	-
**Male/Female**	42/18	21/7	21/11	0.44
**Age (years)**	44.85 ± 10.38	41.71 ± 9.34	47.59 ± 10.59	0.03
**BMI (kg/cm**^**2**^**)**	45.74 ± 8.17	43.73 ± 7.62	47.44 ± 8.34	0.08
**WH-ratio**	1.01 ± 0.15	0.98 ± 0.14	1.05 ± 0.15	0.10
**Glucose (mmol/l)**	6.40 ± 1.87	5.57 ± 0.67	7.13 ± 2.24	<0.001
**Insulin (mU/l)**	18.24 ± 10.39	15.83 ± 7.07	20.56 ± 12.51	0.09
**HbA1c**	6.56 ± 1.31	6.19 ± 0.92	6.93 ± 1.54	0.04
**Total cholesterol (mmol/l)**	4.96 ± 0.98	4.80 ± 0.98	5.10 ± 0.98	0.27
**HDL (mmol/l)**	0.96 ± 0.36	0.99 ± 0.29	0.94 ± 0.43	0.59
**LDL (mmol/l)**	3.11 ± 0.84	2.99 ± 0.92	3.21 ± 0.76	0.35
**TG (mmol/l)**	2.08 ± 1.25	1.87 ± 0.97	2.28 ± 1.47	0.23
**FFA (g/l)**	0.62 ± 0.30	0.53 ± 0.27	0.70 ± 0.31	0.04
**ALAT (U/l)**	27.16 ± 16.92	24.79 ± 10.45	29.44 ± 21.36	0.30
**ASAT (U/l)**	23.28 ± 12.07	19.79 ± 9.86	26.66 ± 13.18	0.03
**CRP (mg/l)**	9.29 ± 6.22	9.91 ± 6.49	8.68 ± 6.00	0.46

MHO, metabolically healthy obese; MUO, metabolically unhealthy obese; BMI, body mass index; WH-ratio, Waist-hip ratio; HbA1c, glycated hemoglobin; HDL, high-density lipoprotein; LDL, low-density lipoprotein; TG, triglyceride; FFA, free fatty acids; ALAT, alanine transaminase; ASAT, aspartate transaminase; CRP, c-reactive protein.

### Inter-tissue co-expression focussing on individual genes

Dobrin *et al*. [25] showed that investigating inter-tissue co-expression can lead to the detection of genes that are related to tissue-specific changes in diseases and that this is representing cross-tissue communication. Therefore, we identified genes that are altered in inter-tissue co-expression between MHO and MUO individuals, as they might pinpoint to genes involved in the crosstalk of tissues with respect to obesity-induced comorbidities. We detected two altered genes between the MHO and MUO subnetwork: *IL1B* and *IL-6*. Both genes showed, most of them significant, stronger inter-tissue co-expression in the MUO subnetwork than in the MHO subnetwork.

Interleukin 1-β (*IL1B*) is an important cytokine mainly produced by activated macrophages. *IL1B* showed a strong correlation between liver and adipose tissues in the MUO subnetwork, with lower correlations among the other tissues and in the MHO subnetwork (Table 2). As obesity leads to an increase of macrophage activation in liver and adipose tissue [26], IL1B mRNA levels will consequently increase. Studies have shown the importance of *IL1B* in the development of obesity-induced insulin resistance [27, 28], and a central role for *IL1B* has been suggested in macrophage-adipocyte crosstalk-related blocking of insulin action in adipose tissue [29]. Moreover, the function of *IL1B* in the liver-adipose tissue crosstalk has been studied in mice [30]. Recent studies showed that there was no difference in systemic IL1B levels between MHO and MUO individuals [31, 32]. Here, we do not find differences in mRNA IL1B levels, but the stronger inter-tissue correlations in the MUO subnetwork indicates a potential role for *IL1B* in obesity-induced metabolic diseases.

**Table 2:** Correlation of *IL1B* mRNA expression between liver and adipose tissues in different groups of subjects, with p-values representing the significance of across-tissue correlation.

Tissues	MHO	MUO	Only T2D	Only NASH	T2D & NASH
Liver—SAT	-0.20	0.59***	0.81*	0.68**	-0.14
Liver—VAT	0.07	0.59***	0.63	0.70**	0.07
SAT—VAT	0.02	0.56***	0.80	0.59**	0.39

MHO, metabolically healthy obese; MUO, metabolically unhealthy obese; T2D, type 2 diabetes; NASH, non-alcoholic steatohepatitis

***P-value<0.001,

**P-value<0.05,

*P-value<0.1

A similar pattern across tissues was found for Interleukin-6 (*IL-6*) with strong correlations between liver and adipose tissues (Table 3). *IL-6* is a cytokine and a myokine, meaning it is also secreted by skeletal muscle during contraction [33]. The many functions of *IL-6* include roles in immunological responses and glucose metabolism [34, 35]. *IL-6* has been proposed as independent predictor of T2D [36] and the same study also showed a significant interaction between *IL1B* and *IL-6*. In contrast to *IL1B*, significantly decreased IL-6 mRNA levels were shown in MHO *vs*. MUO individuals [32]. Surprisingly, our data show no significant difference in IL-6 mRNA levels between MHO and MUO individuals.

**Table 3:** Correlation of *IL-6* mRNA expression between liver and adipose tissues in different groups of subjects, with p-values representing the significance of across-tissue correlation.

Tissues	Healthy	Unhealthy	Only T2D	Only NASH	T2D & NASH
Muscle—Liver	-0.12	0.12	0.06	0.27	-0.11
Muscle—SAT	0.02	0.35**	0.35	0.47*	0.26
Muscle—VAT	0.18	0.35**	0.28	0.44	0.53*
Liver—SAT	0.12	0.46**	0.66	0.47*	0.42
Liver—VAT	-0.07	0.31*	-0.47	0.62**	0.04
SAT—VAT	0.25	0.44	0.32	0.48*	0.58**

MHO, metabolically healthy obese; MUO, metabolically unhealthy obese; T2D, type 2 diabetes; NASH, non-alcoholic steatohepatitis

***P-value<0.001,

**P-value<0.05,

*P-value<0.1

### Inter-tissue network analysis with a focus on *IL1B* and *IL-6*

We constructed two large gene co-expression networks using insulin-related genes in four tissues, resulting in a network for the MHO and for the MUO individuals. Clustering within those networks resultantly can lead to tissue-specific clusters or clusters with genes across tissues. Clustering of the genes in the MHO and MUO network is visualized using a gene dendrogram (S1 Fig). We focussed on the network characteristics of the identified genes (*IL1B* and *IL-6*) as these are potentially important in disease development. *IL1B* and *IL-6*, expressed in each of the four tissues, did not cluster together across tissues or within the MHO subnetwork. However, they did cluster together in the MUO network, within each tissue. We further investigated those modules and genes, focussing on altered correlations between MHO and MUO subnetworks, to get more insight into the mechanisms involved in disease development. We confirmed that those modules were not age and/or gender dependent, as the module eigengenes of the modules were not significantly correlated with age or gender. While we focused on overall co-expression patterns across tissues in MHO ad MUO individuals, it could be expected that co-expression patterns may differ among patients suffering from either NASH or T2D, however, due to the limited sample size we were not able to home in on them.

The Greenyellow module in the MUO network, containing both the *IL1B* and *IL-6* gene, clustered the expression profiles of 29 genes (MM > 0.6) of which 27 are coming from the liver. Two genes in this module (*PKM* and *SLC6A5*) are coming from SAT and VAT, respectively. GSEA revealed the NOD-like receptor (NLR) signalling pathway as most significant KEGG pathway (P = 0.002) and GO-terms were associated with signal transduction (e.g. negative regulation of signal transduction, P = 3.55E^-5^) and cytokine receptor binding (P = 6.59E^-4^). Those findings are in concordance with the fact that excessive amounts of adipose tissue result in an increased release of cytokines, e.g *IL-6*, *IL1B*, and *CCL2*, causing inflammation through, for example, the NLR signalling pathway [37].

**Fig 2:**
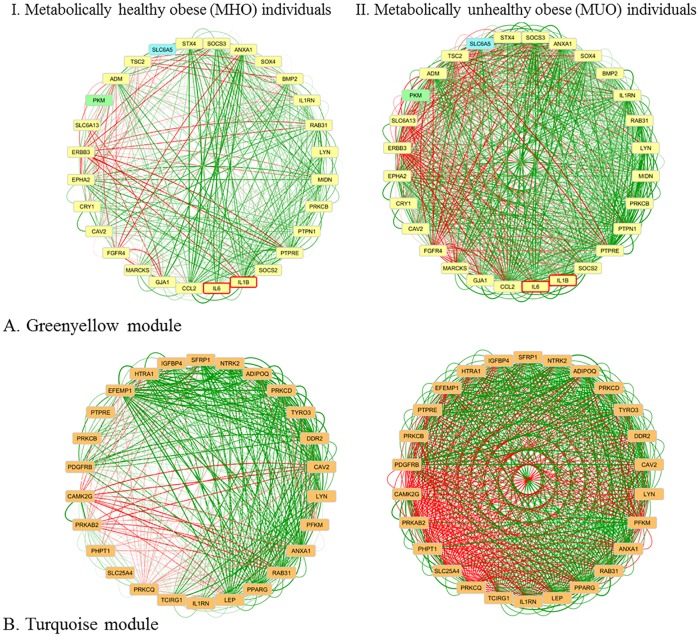
Network visualization of inter-tissue network modules. Visualization of the genes in the (A) Greenyellow module (from MUO subnetwork) in the MHO and MUO individuals, and (B) Turquoise module (from MUO subnetwork) in the MHO and MUO individuals. Genes coming from liver are coloured yellow, from muscle orange, from VAT blue and from SAT green. IL1B and IL-6 are bordered red. Edges are coloured based on their correlation on a red-green scale representing a negative-positive correlation.

Pearson’s correlation coefficients of genes in the Greenyellow module were visualized in Cytoscape for both sub-networks (Fig 2A). Visualization shows that genes in the Greenyellow module are strongly correlated with each other in the MUO subnetwork, while the same genes are less strongly correlated in the MHO subnetwork. As expected, strong co-expression is detected between *IL1B* and *IL-6*, with a slight increase in strength between MHO and MUO individuals (0.50 vs. 0.71, respectively). In this module, *IL1B* does not show large alterations in co-expression with other genes between MHO and MUO individuals. However, *IL-6* shows and altered correlation with the cytokine suppressor genes *SOCS2* (r = 0.01 *vs*. r = 0.74) and *SOCS3* (r = 0.47 *vs*. r = 0.75), and furthermore, the correlation between *SOCS2* and *SOCS3* is slightly altered (r = 0.44 *vs*. r = 0.69). Previous studies have shown that obesity impairs JAK-STAT3 signalling as a result of elevated IL-6 levels, leading to elevated expression of for example *SOCS3* in white adipose tissue, liver, and muscle [38]. By binding to insulin receptor substrates, *SOCS3* impairs insulin action and elevated *SOCS3* levels are associated with insulin resistance [39]. The importance of the association between *SOCS3* and *IL-6* has been shown before, as the absence of *SOCS3* leads to altered effects of *IL-6* resulting in a strong inhibiting effect on macrophages and dendritic cells, resembling an inflammatory response [40]. Also the *PTPN1* gene, encoding PTP1B, is associated with insulin resistance by negatively regulating insulin signalling [41]. Due to its effects on both insulin and leptin, it has been suggested as drug target for obesity and diabetes [42]. This gene showed a highly altered co-expression with *IL-6* in the module between MHO and MUO subnetworks: no correlation was found between *PTP1B* and *IL-6* in the MHO subnetwork, while they were moderately correlated in the MUO subnetwork (r = 0.53).

Detection of potential causal genetic variants using an eQTL mapping approach led to the detection of a noteworthy eQTL in this module: the *SLC6A5* gene, expressed in VAT. *SLC6A5* shows generally low correlation coefficients with other genes in the MHO subnetwork, but several strongly correlated genes in the MUO subnetwork. Correlations with *IL1B* and *IL-6* are only moderate in MUO (0.28 and 0.41, respectively) and low in MHO (-0.13 and -0.05, respectively) subnetworks. The strongest correlations of *SLC6A5* within the MUO subnetwork are found with *ANXA1* (r = 0.66), *CCL2* (r = 0.63), *MARCKS* (r = 0.61), and *SOCS3* (r = 0.61); all of which are not correlated in the MHO subnetwork. *SLC6A5* is encoding the neuronal glycine transporter 2 (GlyT2) and its action involves protein kinase C (PKC) pathways [35]. Even though *SLC6A5* itself has not been associated with obesity, its regulating properties via PKC pathways might affect the metabolic state of obese individuals. For example, *ANXA1* (associated with adiposity [43]) is an important mediator in the neuroendocrine system and PKC-dependent mechanisms are essential for its activity [44]. Likewise, the previously discussed *SOCS3* has been linked to involvement in PKC pathways [45]. We therefore suggest a potential causal role for *SLC6A5* in regulatory processes related to obesity-induced metabolic diseases. This is supported by the fact that a mutation in this gene causes, among others, a decreased body weight in mice [46].

The Turquoise module in the MUO subnetwork (Fig 2B) clustered the expression profiles of 26 unique genes (MM > 0.9), all coming from the muscle. Both *IL1B* and *IL-6* were filtered out due to the strict threshold on the MM, but were included in comparison of correlation strengths between the MHO and MUO subnetworks. The correlations among them were altered between the MHO and MUO subnetwork (r = 0.25 *vs* r = 0.75, resp.). One well-known obesity-genes is Leptin (*LEP*), encoding leptin, which is also called the “satiety hormone”. In skeletal muscle, it promotes energy dissipation and prevents fatty acid accumulation [47]. *LEP* shows strong positive and negative correlations with all other Turquoise module-genes (absolute correlation > 0.8) in the MUO subnetwork, while being low with many of the genes in the MHO subnetwork (absolute correlation < 0.4). The largest differential correlation for LEP was found between *LEP* and *PTPRE* (0.22 *vs*. 0.91 in MHO *vs*. MUO). *PTPRE* (Protein Tyrosine Phosphatase, Receptor Type, E) negatively regulates insulin signaling in skeletal muscle and has been suggested to play a role as negative feedback regulator of leptin signaling via JAK2 [8]. Furthermore, strong alterations were found for the correlation between *LEP* and *IL1B* (r = -0.15 *vs*. r = 0.81 in MHO *vs*. MUO). It has been shown that *IL1B* is necessary for induction of leptin during inflammation [12]. Our data suggest that this induction is not occurring in MHO individuals. Another interesting gene is Adiponectin (*ADIPOQ*), encoding the hormone adiponectin that enhances skeletal muscle insulin sensitivity and has been suggested as a drug target for obesity and T2D [13]. The correlation between *ADIPOQ* and *IL1B* differed to a large extent between MHO and MUO (r = -0.19 *vs* r = 0.68 in MHO *vs*. MUO). In adipocytes, but not in skeletal muscle, it has been shown that *IL1B* reduces adiponectin production, thereby negatively affecting insulin sensitivity [24, 29]. No eQTLs were detected in this module.

**Fig 3:**
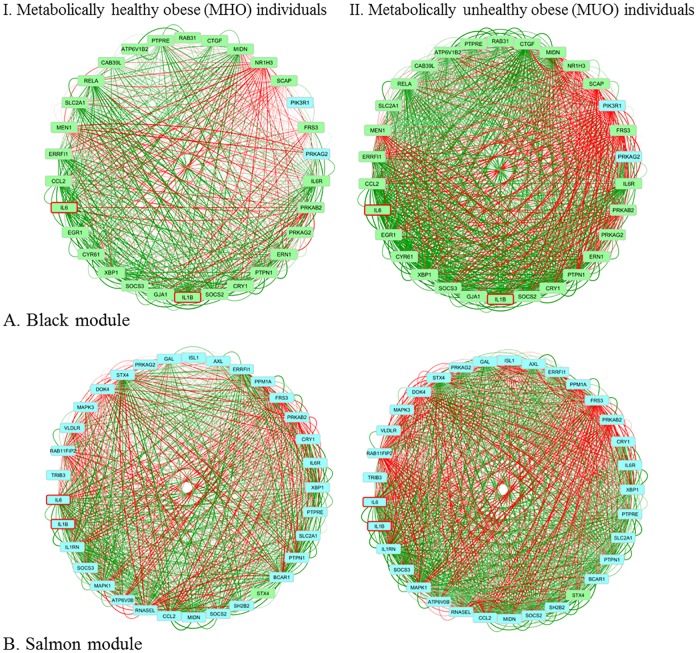
Network visualization of the inter-tissue network modules. Visualization of the genes in the (A) Black module (from MUO subnetwork) in the MHO and MUO individuals, and (B) Salmon module (from MUO subnetwork) in the MHO and MUO individuals. Genes coming from liver are coloured yellow, from muscle orange, from VAT blue and from SAT green. IL1B and IL-6 are bordered red. Edges are coloured based on their correlation on a red-green scale representing a negative-positive correlation.

The Black module in the MUO subnetwork (Fig 3A) clustered the expression profiles of 30 genes (MM < 0.60), all coming from SAT, except for two that were coming from VAT: *PRKAG2* (also co-expressed in SAT) and *PIK3R1*. The correlation between *IL1B* and *IL-6*, and their correlation with cytokine suppressor genes, was similar between MHO and MUO subnetworks in SAT. The two genes that are expressed in VAT, *PRKAG2* and *PIK3R1*, are showing altered co-expression with *IL-6* and *IL1B*. The co-expression of *PIK3R1* with *IL1B* and *IL-6* in the MHO subnetwork is very low (-0.16 and -0.20, respectively), but strongly negative in the MUO subnetwork (both -0.67). *PIK3R1* encodes part of the phosphatidylinositol 3-kinase (PI3K) enzyme, and PI3K signaling plays an important role in insulin signaling. *PIK3R1* is involved in the mediation of insulin sensitivity and the inflammatory response in adipose tissue [31]. The altered co-expression between the insulin genes and *PIK3R1* indicates an important role in disease development that has been identified by others as well [48]. The correlation of *PRKAG2* with *IL1B* and *IL-6* is around zero in the MHO, but moderately positive in the MUO subnetwork (0.53 and 0.54, respectively). *PRKAG2* is part of the AMP-activated protein kinase (AMPK) pathway, which is important in energy regulation, e.g. glucose homeostasis and insulin sensitivity [49]. Due to its properties, it is a drug target for metabolic syndrome.

In this module we found one eQTL: the IL-6 receptor (*IL-6R*). Many of the correlations between *IL-6R* and other genes were not altered between MHO and MUO subnetworks. However, the correlation with *SOCS3* was elevated (0.29 vs 0.67, respectively) and with *PIK3R1* (in VAT) was decreased (0.01 vs -0.58, respectively). Surprisingly, the correlations of *IL-6* with those genes were unaltered, implicating an important role for the receptor of IL-6, also based on previous discussion of results for the JAK-STAT pathway, in obesity-induced disease development. Several altered phenotypes have been detected in mutations in the IL-6 receptor in mice, e.g. insulin resistance and inflated inflammatory action [50]. However, in a large human survey, a significant correlation with BMI and weight loss within morbidly obese individuals could only be found with IL-6R expression levels in liver, and not in omental adipose, subcutaneous adipose and stomach tissue [51].

The Salmon Module in the MUO subnetwork (Fig 3B) clustered the expression profiles of 33 genes (MM < 0.6). These genes were all coming from VAT, except for one gene (Syntaxin 4, *STX4*) that was coming from both SAT and VAT. Several genes from previously discussed modules were also present in this module. The co-expression of *IL1B* and *IL-6* with *SOCS2* and *SOCS3* is similar to the co-expression found in SAT (Black module), meaning that their correlation is only altered in the liver. Also the co-expression between *IL1B* and *IL-6* is unaltered in SAT. The co-expression between *STX4* in SAT and VAT is stronger in the MUO than in the MHO subnetwork (0.28 *vs* 0.58, respectively). The co-expression of *STX4* in SAT is altered with *IL1B*, the IL1B receptor antagonist (*IL1RN*), *IL6*, and the IL-6 receptor (*IL6R*). In all four cases, the co-expression is moderate to strongly positive in the MUO subnetwork and low negative co-expressed in the MHO subnetwork. However, the co-expression of *STX4* in VAT with the four genes that were expressed in VAT is similar between MHO and MUO subnetworks, suggesting a role in disease development for *STX4* only in SAT.

In this module we found one eQTL: *PTPRE*, expressed in VAT. *PTPRE* is a negative regulator of insulin signalling in muscle [47]. In a study with obese individuals, it was shown that *PTPRE* was differentially expressed and methylated before and after bariatric surgery, and that NASH phenotype was negatively correlated with the bariatric reconstruction [52]. Besides the detection of *PTPRE* as eQTL, the *PTPRE* gene is present in all four modules that were further investigated due to presence of *IL1B* and *IL-6*. Homing in on the cross-tissue talk of this gene, the main change is found between the correlation in liver and VAT (0.05 vs 0.55 in MHO vs. MUO). In the Salmon module *PTPRE* shows altered co-expression with the previously discussed *ISL1* gene (0.07 vs -0.60 in MHO vs MUO), encoding the insulin gene enhancer protein ISL-1. Many of the correlations of *ISL1* with other genes in this module are altered, with strong negative correlations in the MUO subnetwork. Previously, it has been shown that *ISL1* is expressed in VAT and negatively correlated with BMI and abdominal fat [34]. They also showed that expression was reduced in obese mice but reduced in lean insulin sensitive mice. In the Greenyellow module, with mostly liver genes, alterations between MHO and MUO individuals are found within the correlations of the expression of *PTPRE* in liver with e.g. *SLC6A5* (VAT), *SLC6A13* (liver) and *PKM* (SAT). It is remarkable that the module consists of mostly genes co-expressed in the liver, but that *PTRPE* shows altered co-expression with the two genes that are coming from SAT and VAT, suggesting an important function for *PTPRE* as a link between tissues in disease development resulting from obesity. To date, no studies have linked *PTPRE* with any of those genes.

In summary, we have shown the integration of gene co-expression networks across tissues in MHO and MUO individuals to identify genes and pathways related to obesity-induced disease development. The results provide important insights into genomics of adipose tissue expandability, lipotoxicity, and eventual comorbidities such as T2D and NASH. In a co-expression network setting, we detected *IL-6* and *IL1B* as key genes for inter-tissue gene co-expression differences related to metabolic state. By investigating the modules in which *IL-6* and *IL1B* were co-expressed, we detected many altered co-expressed genes and pathways that might be important in obesity-induced inflammation and comorbidity development. Even though *IL-6* and *IL1B* mRNA levels were not altered between MHO and MUO individuals in our study, their co-expression with other genes indicates a potentially important role in obesity-induced development of metabolic disturbances. The chosen approach gave us insight into co-expression between genes in a network setting, but could not give information about directions in the network. However, an eQTL mapping approach was chosen to detect genes that affect mRNA levels and thereby affecting health status. This led to the identification of several genes (*PTPRE*, *IL-6R*, and *SLC6A5)* which might have an important role in insulin-related pathways of obese individuals. Functional validation of those genes is needed to identify their potential role in the development of obesity-induced comorbidities.

## Supporting Information

S1 Fig Clustering of the insulin-related genes. Clustering is based on the dissimilarity Topological Overlap Measure (TOM) within the A) metabolically healthy obese (MHO) and B) metabolically unhealthy obese (MUO) network. Modules are detected using the Dynamic Tree Cut algorithm and presented by the color-coded bar under the dendrogram.
(PNG)
