# Supplementary figures and images for: Inter-Tissue Gene Co-Expression Networks between Metabolically Healthy and Unhealthy Obese Individuals
Source: PLoS One. 2016 Dec 1;11(12):e0167519. doi: 10.1371/journal.pone.0167519 (PMC5132173; doi:10.1371/journal.pone.0167519)

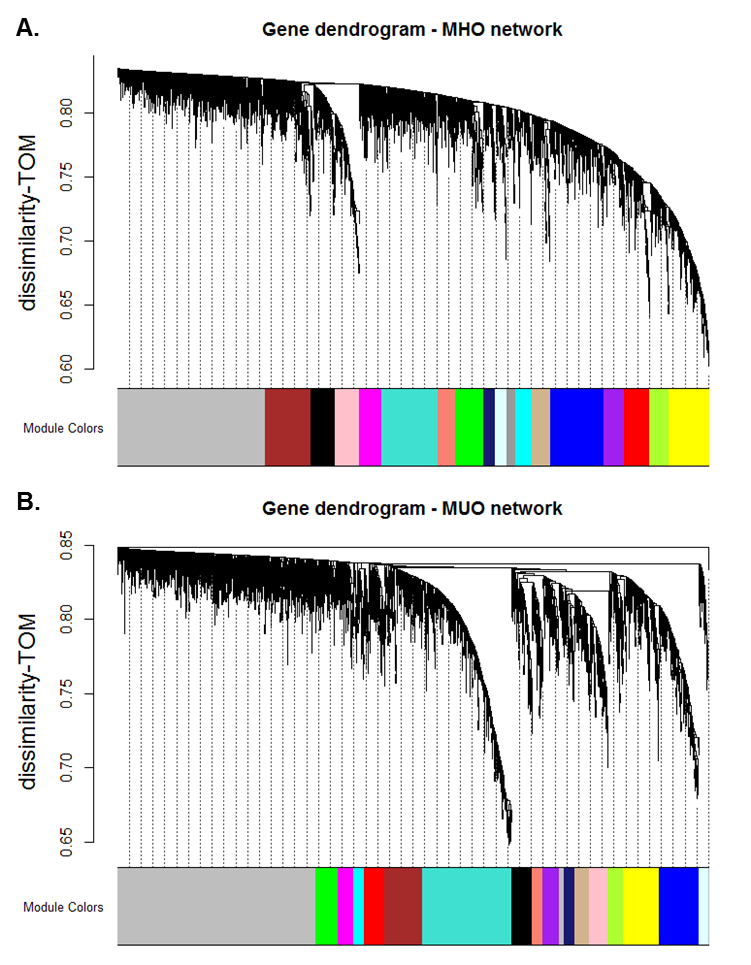

Supplement: S1 Fig — Clustering is based on the dissimilarity Topological Overlap Measure (TOM) within the A) metabolically healthy obese (MHO) and B) metabolically unhealthy obese (MUO) network. Modules are detected using the Dynamic Tree Cut algorithm and presented by the color-coded bar under the dendrogram. (PNG) [file pone.0167519.s001.png]
